# Involvement of circRNAs in Proinflammatory Cytokines-Mediated *β*-Cell Dysfunction

**DOI:** 10.1155/2021/5566453

**Published:** 2021-05-04

**Authors:** Zhen Wang, Chao Deng, Ying Zheng

**Affiliations:** ^1^Department of Metabolism and Endocrinology, The Second Xiangya Hospital, Central South University, Changsha, 410011 Hunan, China; ^2^Key Laboratory of Diabetes Immunology (Central South University), Ministry of Education, National Clinical Research Center for Metabolic Diseases, Changsha, 410011 Hunan, China; ^3^Center for Medical Research, The Second Xiangya Hospital, Central South University, Changsha, 410011 Hunan, China

## Abstract

**Aim:**

During the initial stage of type 1 diabetes, prolonged exposure of pancreatic *β*-cell to proinflammatory cytokines such as IL-1*β*, TNF-*α*, and IFN-*γ* results in a decreased capacity to produce and release insulin, as well as cell loss by apoptosis. Circular RNAs (circRNAs) are a new class of endogenous noncoding RNAs (ncRNAs) with closed loop with no free ends. circRNAs have been reported to be participated in the development of many diseases. As little is known about their role in insulin-secreting cells, this study is aimed at evaluating their contribution in the process of inflammation-induced *β*-cell damage.

**Methods:**

circRNA expression profile of MIN6 cells stimulated with a mix of cytokines, including IL-1*β*, IFN-*γ*, and TNF-*α*, was detected by circRNA microarrays. Four dysregulated circRNAs were validated by qRT-PCR. The involvement of the selected circRNAs in *β*-cell dysfunction was tested after their inhibition in MIN6 cells. MicroRNA target prediction software and multiple bioinformatic approaches were used to predict the targeting genes of circRNAs and analyze possible functions of the circRNAs.

**Results:**

1020 upregulated and 902 downregulated circRNAs were identified in cytokines-treated *β*-cells. Inhibition of circRNAs 000286 and 017277 in *β*-cells could promote *β*-cell apoptosis and affect insulin biosynthesis and secretion. GO analysis enriched terms such as regulation of transcription and regulation of gene expression and KEGG analysis enriched top pathways included TGF-*β* and MAPK signaling pathways.

**Conclusions:**

The data shows that circRNAs may be involved in proinflammatory cytokines-mediated *β*-cell dysfunction and suggests the involvement of circRNAs in the development of type 1 diabetes.

## 1. Introduction

Circular RNAs (circRNAs) were newly discovered as a novel class of endogenous noncoding RNAs (ncRNAs) [1–3]. Unlike linear RNAs terminated with 5′ caps and 3′ tails, circRNAs form covalently closed loop with no free ends [2, 4]. circRNAs are generated from exons, introns, or both, even the tRNA introns [5–8]. Up to now, circRNAs are known to be widely expressed in many tissues and eukaryotic organisms and function in multiple biological and pathological processes [2, 5, 9, 10]. The most frequent discovers were that circRNAs act as microRNA (miRNA) sponges to inhibit miRNA function [11]. For instance, ciRS-7/CDR1as harbors >70 conserved binding sites for miR-7, which activity could be dramatically reduced through tethering of this miRNA to ciRS-7/CDR1as [12]. Additionally, circRNAs can interact with RNA-binding proteins to regulate protein function [13]. Recent studies also showed that the formation of circRNAs can affect the generation of liner RNAs [13]. As well as these validated functions, several other putative functions have been suggested for circRNAs, including transport vehicles and long-term sensors [14]. Since the wide range of roles, it is not surprising that circRNAs play an important role in many biological and pathological processes. And the dysregulation of circRNAs will contribute to the pathogenesis of many diseases, such as cancers, neurodegenerative diseases, and autoimmune diseases [15, 16].

A good control of appropriate amount of insulin released by pancreatic *β*-cells is essential to maintain the blood glucose homeostasis. Type 1 diabetes is a chronic autoimmune disease characterized by inflammatory response against pancreatic islets, consequently resulting in progressive *β*-cell destruction and defective insulin secretion [17, 18]. During the initial stage of type 1 diabetes, *β*-cells are exposed to the proinflammatory cytokines, such as IL-1*β*, IFN-*γ*, and TNF-*α*, released by the infiltrated immune cells [17–20]. And the inflammatory microenvironment has deleterious effects on *β*-cells, leading to impaired insulin production and secretion as well as *β*-cell apoptosis. Therefore, a better understanding of pathological events and regulatory mechanisms involved in proinflammatory cytokines-mediated *β*-cell destruction will be helpful to clarify the pathogenesis of type 1 diabetes and develop new approaches for preventing and treating the disease. There is already strong evidence indicating several groups of ncRNAs, including miRNAs and long noncoding RNAs, as novel players in regulating *β*-cell functions during the process of type 1 diabetes [21–28]. Indeed, circRNAs may also be important regulators of specialized *β*-cell functions. ciRS-7 has been reported to play important roles in the control of *β*-cell functions and be dysregulated under diabetic conditions [12]. In addition, Stoll et al. have shown that circHIPK3 and ciRS-7 are highly abundant in pancreatic islets and display reduced expression in diabetic animal models, and silencing these circular transcripts results in impaired *β*-cell function, pointing to a contribution of altered circHIPK3 and ciRS-7 to the development of diabetes mellitus [29]. Recently, circular RNA HIPK3 has been demonstrated to contribute to hyperglycemia and insulin homeostasis by sponging miR-192-5p, subsequently upregulating transcription factor forkhead box O1 [30].

In the present study, we investigated the involvement of circRNAs in cytokine-mediated *β*-cell damage and in the development of type 1 diabetes. We found that exposure of *β*-cell to proinflammatory cytokines could result in changed expression of a subset of circRNAs, which might alter insulin biosynthesis and secretion and affect *β*-cell apoptosis. Furthermore, we analyzed the possible regulatory mechanisms of some circRNAs in cytokines-mediated *β*-cell destruction.

## 2. Materials and Methods

### 2.1. Cell Culture and Chemicals

The insulin secreting cell line MIN6 was cultured in Dulbecco's modified Eagle's medium (DMEM, Invitrogen) supplemented with 10% heat-inactivated fetal calf serum (FBS, Gibco, Thermo Fisher), 50 IU/mL penicillin, 50 mg/mL streptomycin, and 70 mmol/L *β*-mercaptoethanol (Millipore). The cells were maintained at 37°C in a humidified atmosphere of 5% CO_2_. Recombinant mouse interleukin-1*β* (IL-1*β*) was purchased from Sigma. IFN-*γ* and TNF-*α* were purchased from R&D Systems. Hoechst dye 33342 was purchased from Beyotime (Jiangshu, China).

### 2.2. Microarray and RNA Sequencing Profiling

MIN6 cells were seeded into 6-well plate at approximately 80% confluence and were then treated with or without a mix of 2.5 ng/mL IL-1*β*, 2.5 ng/mL TNF-*α*, and 25 ng/mL IFN-*γ* for 24 h. The total RNA was extracted from the cells by using Trizol reagent (Ambion). For circRNA profiling, RNA was treated with RNase R to deplete the linear transcripts and enrich circular RNA. circRNA expression profiling and data analysis were carried out using Arraystar Mouse circRNA Array V2 (Kangchen Inc, Shanghai, China). The raw data from the circRNA microarray was normalized by the GeneSpring software (V. 12.5). Differentially expressed circRNAs were identified with *P* value <0.05 and fold‒change ≥ 1.5 and further analyzed with hierarchical clustering (HCL).

### 2.3. Quantitative Real-Time PCR

Total RNA isolated from MIN6 cells was treated with RQ1 RNase-free DNase and reversely transcribed using a reverse reaction kit, according to the manufacturer's instructions (Promega). For mRNA amplification, the total RNA was reversely transcribed with oligo-dT primer, while random primer sets were used for circRNA amplification. The qPCR was performed with a SYBRGreen supermix (Takara, Dalian, China) on a Roche PCR System (Roche, Basel, Switzerland). The relative expression was normalized to the level of a housekeeping gene, GAPDH. The circRNA expression was calculated by using 2^-*ΔΔ*Ct^ method. All the results are the average ratios of three different independent experiments. Primer sequences are listed in Table 1.

### 2.4. circRNA siRNA Transfection

To knockdown circRNA, the MIN6 cells were seeded into 6-wellplate and transfected with circRNA siRNAs (GenePharma, Shanghai, China). An unrelated known siRNA was used as a control. The transfections of MIN6 cells were performed with Lipofectamine 2000 (Invitrogen) according to the manufacturer's protocols.

### 2.5. Insulin Content and Secretion Determination

The MIN6 cells were seeded into 6-well plate and transfected with circRNA siRNAs. After 48 h, the cells were washed and preincubated for 45 min in Krebs buffer (127 mmol/L NaCl, 4.7 mmol/L KCl, 1 mmol/L CaCl_2_, 1.2 mmol/L KH_2_PO_4_, 1.2 mmol/L MgSO_4_, 5 mmol/L NaHCO_3_, 0.1% BSA, and 25 mmol/L HEPES, pH 7.4) containing 2 mmol/L glucose. Then, the cells were lysed by 75% ethanol and 0.55% HCl to determine total insulin content. The amount of insulin in the cells was measured by ELISA. For glucose stimulated insulin secretion (GSIS), the cells were incubated in a Krebs buffer containing 20 mmol/L glucose. After 45 min stimulation, supernatants were collected. The amount of insulin in the samples was determined by ELISA.

### 2.6. Cell Death

The MIN6 cells were seeded in 24-well plate and transfected with circRNA siRNAs. After 3 days, the cells were stained with Hoechst 33342 (Bytiome, Wuhan, China), and fraction of dying cells was determined by scoring the cells displaying picnotic nuclei upon the staining. The experiments were performed in duplicates and repeated at least for three times.

### 2.7. circRNA-miRNA-mRNA Network Analysis

To predict the functions of circRNAs, the circRNA-miRNA-mRNA network was constructed according to Arraystar's in-house miRNA target prediction software based on TargetScan and miRDB. The circRNA-miRNA-mRNA interaction network was visualized by using the Cytoscape software (V. 3.2.1).

### 2.8. Gene Ontology (GO) and Kyoto Encyclopedia of Genes and Genomes (KEGG) Pathway Analysis

The predicted circRNA-related mRNAs were selected for GO term and KEGG pathway analysis by using the Database for Annotation, Visualization, and Integrated Discovery (DAVID). The -log10 (*P* value) indicated the enrichment score, which represents the significance of GO term and KEGG pathway enrichment.

### 2.9. Statistical Analysis

The statistical analyses were conducted by using the SPSS 15.0 software and GraphPad Prism 5. The results are presented as mean ± SD from three independent experiments. Data was analyzed by Student's *T* or ANOVA test. *P* < 0.05 was considered as being statistically significant.

## 3. Results

### 3.1. Changed Expression Profile of circRNAs in Cytokines-Mediated Pancreatic β-Cell Destruction

**Figure 1:**
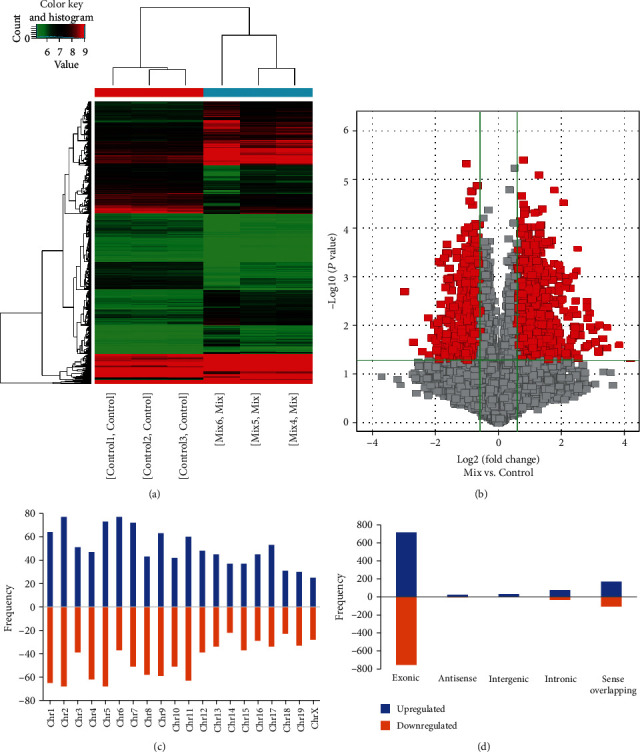
Changed expression profiles of circRNAs in cytokines-mediated pancreatic-*β* cell destruction. (a) The heat map of differentially expressed circRNAs in cytokines-treated pancreatic-*β* cells and control cells from microarray data. (b) Scatter plots showing differentially expressed circRNAs in cytokines-treated pancreatic-*β* cells and control cells. (c) Chromosomal frequency distribution of differentially expressed circRNAs. (d) Types of differentially expressed circRNAs. The circRNAs were classified into five types including exonic, antisense, intergenic, intronic, and sense overlapping. Blue and orange indicate upregulated and downregulated circRNAs, respectively.

To investigate the potential involvement of circRNAs in *β*-cell destruction occurring during the initial stage of type 1 diabetes, the mouse insulin-secreting cell line MIN6 cells were treated with a mix of cytokines, 2.5 ng/mL IL-1*β*, 2.5 ng/mL TNF-*α*, and 25 ng/mL IFN-*γ* for 24 h. The expression profile of circRNAs was determined by Arraystar Mouse circRNA Array V2, which can detect circRNAs. Hierarchical clustering showed a distinguishable circRNA expression profile in cytokines-treated MIN6 cells compared with controls. Upon the treatment with cytokines, 1020 circRNAs were upregulated, and 902 were downregulated with a set filter fold change ≥ 1.5 (Figures 1(a) and 1(b)). All the differentially expressed circRNAs were statistically significant (*P* < 0.05). The distribution of the differentially expressed circRNAs on mouse chromosomes was shown in Figure 1(c). It suggests that each chromosome is related to a varying frequency of differentially expressed circRNAs. We also analyzed the characteristics of the differentially expressed circRNAs according to the genomic loci and their relationship with the associated coding genes. They were classified into five types: exonic, antisense, intergenic, intronic, and sense overlapping (Figure 1(d)). The results showed that most of the differentially circRNAs were transcribed from the protein-coding exons (Figure 1(d)).

### 3.2. Validation for the Expression of Significant circRNAs by qRT-PCR

**Figure 2:**
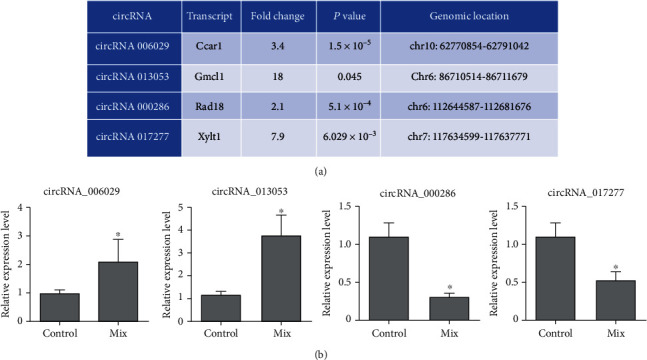
Validation for the expression of four dysregulated circRNAs. (a) The four dysregulated circRNAs were selected and shown with fold changes, *P* values, and genomic locations. (b) The expression of the four selected circRNAs were changed by proinflammatory cytokines. MIN6 cells were incubated in the absence or presence of 2.5 ng/mL IL-1*β*, 2.5 ng/mL TNF-*α*, and 25 ng/mL IFN-*γ* (24 h incubation). The expression levels were measured by qRT-PCR. The results are shown as means ± SD for three independent experiments. ^∗^*P* < 0.05, compared with control group.

Two upregulated, circRNAs 006029 and 013053, and two downregulated circRNAs, circRNAs 000286 and 017277, were selected for further analysis. The fold changes of these circRNAs in response to cytokine treatment and their genomic location are displayed in Figure 2(a). Next, the expression levels of the selected dysregulated circRNAs were validated by qRT-PCR. As shown in Figure 2(b), circRNAs 006029 and 013053 were upregulated, while circRNAs 000286 and 017277 were downregulated, which was consistent with the microarray data.

### 3.3. The Effect of circRNAs 000286 and 017277 on Insulin Synthesis and Secretion

**Figure 3:**
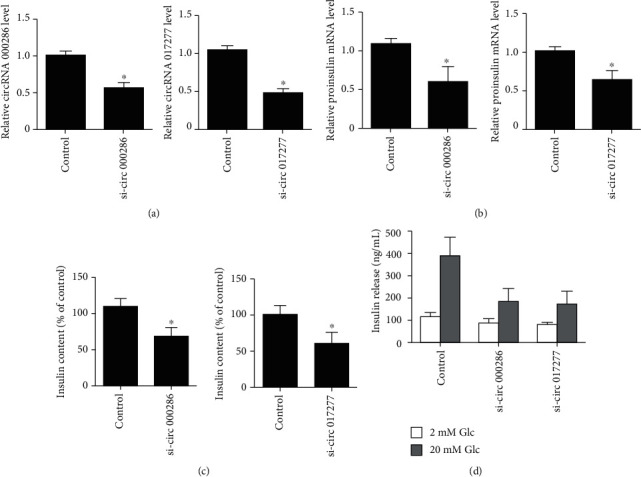
Effect of circRNAs 000286 and 017277 on insulin biosynthesis and secretion in MIN6 cells. (a) The MIN6 cells were transfected with circRNA 000286 or 017277 siRNAs. The total RNA was extracted, and the expression levels of circRNA 000286 (left panel) or circRNA 017277 (right panel) were determined to evaluate the efficiency of transfection. The results are mean ± SD of three independent experiments. (b) The MIN6 were transfected with circRNA 000286 (left panel) or circRNA 017277 (right panel) siRNAs or negative control. Proinsulin mRNA levels were measured 36 h later by qRT-PCR, and the results are expressed normalized to GAPDH. (c) The MIN6 were transfected with circRNA 000286 (left panel) or circRNA 017277 (right panel) or negative control. Insulin content was assessed by ELISA, and the results are expressed as percent of the values in control cells. The data shown are the mean ± SD of three independent experiments. (d) The MIN6 were transfected with circRNA 000286 or 017277 siRNAs or negative control. Two days later, the cells were incubated in the presence of 2 or 20 mmol/L glucose (Glc) for 45 min. The amount of insulin secreted during the incubation period was assessed by ELISA assays. The data shown are the mean ± SD of three independent experiments. ^∗^*P* < 0.05, compared with respective control.

Then, we determined whether the changed circRNAs could affect specific *β*-cell functions. We firstly inhibited the expression of circRNAs 000286 and 017277 by transfecting circRNA siRNAs (Figure 3(a)) and then assessed the impact of the two circRNAs on insulin biosynthesis. The results showed that inhibition of circRNAs 000286 and 017277 led to a reduction of proinsulin mRNA levels and total insulin content in MIN6 cells (Figures 3(b) and 3(c)). We also detected the effect of circRNA 000286 and 017277 inhibition on insulin secretion. As shown in Figure 3(d), circRNA 000286 and 017277 inhibition could inhibit the capacity of GSIS of pancreatic *β*-cell.

### 3.4. The Effect of circRNAs 000286 and 017277 on *β*-Cell Apoptosis

**Figure 4:**
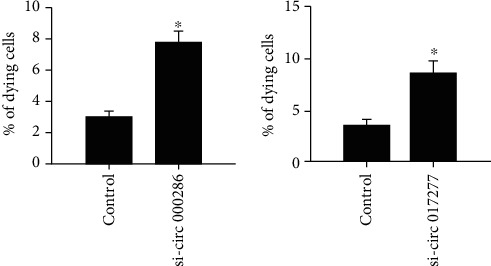
Effect of circRNAs 000286 and 017277 on pancreatic-*β* cell apoptosis. The MIN6 were transfected with circRNA 000286 (left panel) or circRNA 017277 (right panel) siRNAs or negative control. The fraction of dying cells was determined 3 days later by scoring the cells displaying picnotic nuclei upon Hoechst 33342 staining. The results are the mean of three experiments. ^∗^*P* < 0.05, compared with respective control.

We next investigated the effect of circRNAs 000286 and 017277 changed by the treatment of cytokines on cell apoptosis. As shown in Figure 4, the transfection of circRNA 000286 and 017277 siRNAs in MIN6 cells caused increased number of cells undergoing apoptosis.

### 3.5. Construction of circRNA-miRNA-mRNA Network

**Figure 5:**
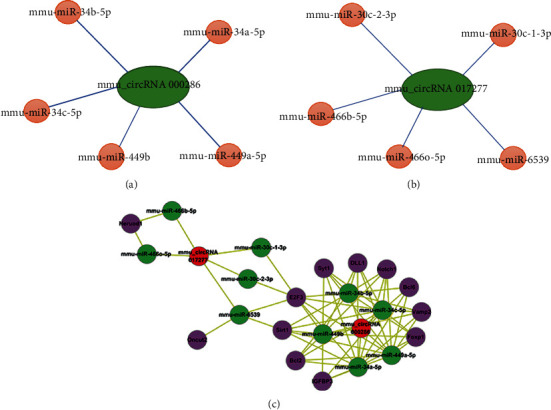
Construction of the specific circRNA-miRNA-mRNA ceRNA regulatory network for circRNAs 000286 and 017277. Predicted miRNAs that may interact with circRNA 000286 (a) and 017277 (b). Green circles represent the selected circRNAs. Orange circles represent the predicted miRNAs. (c) The specific circRNA-miRNA-mRNA ceRNA regulatory network for circRNAs 000286 and 017277. Red circles represent the selected circRNAs. Green circles represent the predicted miRNAs. Purple circles represent the predicted mRNAs.

As one type of ncRNA, circRNAs can function as competing endogenous RNAs (ceRNAs) or natural microRNA sponges by competitively binding to miRNAs, thereby regulating gene expression. Therefore, circRNA-miRNA-mRNA interactions may be an important mechanism underlying the functions of circRNAs 000286 and 017277 in inflammatory cytokines-induced pancreatic *β*-cell dysfunction. Next, we attempted to explore the possible mechanism of circRNAs 000286 and 017277 regulating pancreatic *β*-cell function and apoptosis. Based on the bioinformatic software, the predicted miRNAs that may bind to circRNAs are shown in Figures 5(a) and 5(b). By TargetScan and miRDB, the targeted mRNAs of these miRNAs were also obtained, and then, the circRNA-miRNA-mRNA network was constructed (Figure 5(c)). These RNA interactions may supply a novel perspective for the pathogenesis of pancreatic *β*-cell damage in type 1 diabetes.

### 3.6. GO Terms and KEGG Pathway Enrichment Analysis of circRNAs 000286 and 017277-Related mRNAs

**Figure 6:**
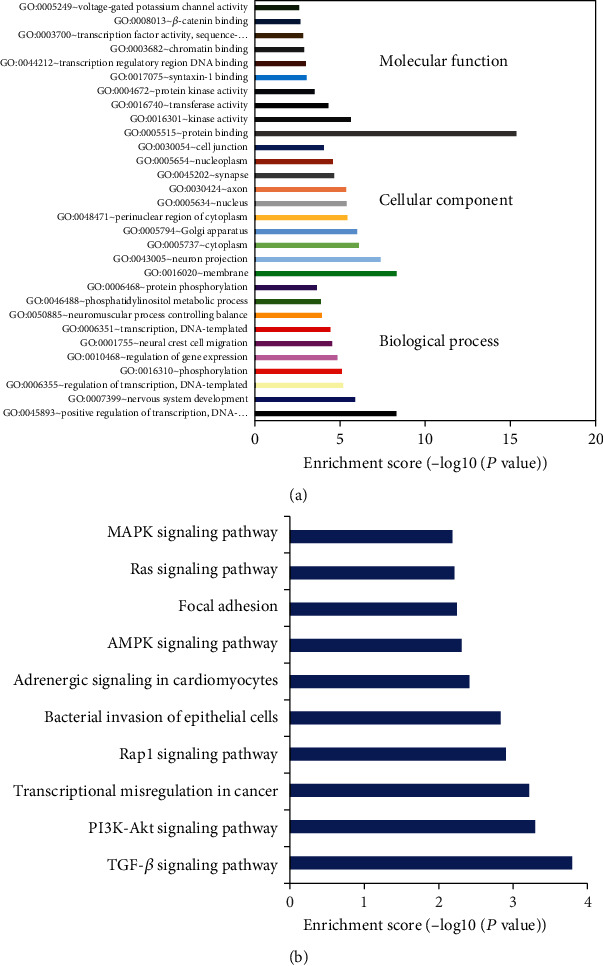
GO terms and KEGG pathway enrichment analysis of circRNA 000286 and 017277-related mRNAs. (a) Top 10 biological process, cellular component, and molecular function of GO terms for the analysis of circRNA 000286 and circRNA 017277-related mRNAs. (b) Top 10 KEGG pathway for analysis of the two circRNAs.

Next, GO term and KEGG pathway analyses were performed on related genes of the two circRNAs by DAVID bioinformatics resources. The GO enrichment analysis was conducted mainly on three domains, namely, biological process (BP), cellular component (CC), and molecular function (MF). GO analysis enriched terms such as protein binding, positive regulation of transcription, regulation of gene expression, and KEGG analysis enriched top pathways included TGF-beta and MAPK signaling pathways (Figures 6(a) and 6(b)).

## 4. Discussion

Type 1 diabetes is characterized by infiltration of the islets of Langerhans by immune cells and selective and progressive destruction of pancreatic *β*-cells [17, 18]. The infiltration of immune cells has been observed both in nonobese diabetic (NOD) mice and in the islets of human diabetic donors. During the initial phases of the disease, the infiltrating immune cells can release cytokines and other proapoptotic mediators, subsequently leading to *β*-cell death and dysfunction. Previous studies on that proinflammatory cytokines exert hazardous effects on *β*-cell often focused on the regulation of protein-coding genes and the changed signaling pathways [19, 20]. In recently years, the roles of ncRNAs in cytokines-induced *β*-cell destruction increasingly draw the attention.

ncRNAs are a vast majority of human RNA transcripts that do not encode proteins, which include miRNA, piRNA, tiRNA, lncRNAs, and circRNAs [31–33]. miRNAs are well-known posttranscriptional regulators of gene expression. Recently, another novel class of ncRNA, circRNA, has also been identified as important regulators of gene expression. They are involved in gene regulation at different levels, from epigenetic gene silencing to posttranscriptional regulation of mRNA stability [2, 3, 14]. circRNAs can regulate cellular functions and are important regulators in many physiological and pathological conditions. Recently, some circRNAs have been reported to be implicated in pancreatic *β*-cell function. circHIPK3 and ciRS-7/CDR1as were found to be reduced in the islets of diabetic db/db mice. Inhibition of circHIPK3 and ciRS-7/CDR1as in the islets of wild type animals resulted in impaired insulin secretion, reduced *β*-cell proliferation, and survival [12]. Circular RNA circPPM1F modulates M1 macrophage activation and pancreatic islet inflammation in type 1 diabetes [34]. Circulating circular RNA profiles have also been reported to be associated with type 1 diabetes [35]. 2600 circRNAs were identified to be present in human islets, and circCIRBP, circZKSCAN, circRPH3AL, and circCAMSAP1 were demonstrated to have marked associations with diabetes status. CircCIRBP was demonstrated to have an association with insulin secretory index in isolated human islets. CircCIRBP and circRPH3AL displayed altered expression with elevated fatty acid in treated EndoC-*β* H1 cells. CircCAMSAP1 was also noted to be associated with type 2 diabetes status in human peripheral blood [36].

In the present study, the expression profiles of circRNAs in mouse pancreatic *β*-cells were detected following the stimulation of cytokine mix, including IFN-*γ*, IL-1*β*, and TNF-*α*. From the results, we figured out that during the process of cytokines-induced pancreatic *β*-cell destruction, 1020 upregulated circRNAs and 902 downregulated circRNAs were observed. circRNAs are classified into five distinct types: antisense, exonic, intergenic, intronic, and sense overlapping. The most type of differentially expressed circRNA identified in this study was exonic. Then, we focused on two downregulated circRNAs and demonstrated that inhibition of circRNA could suppress insulin biosynthesis and secretion and promote the apoptosis of pancreatic *β*-cells. These results suggested that changed circRNAs induced by cytokine stimulation might contribute to cytokines-induced pancreatic *β*-cell dysfunction, and they may have an important role in the onset and development of type 1 diabetes.

One important mechanism that circRNAs regulate gene expression is that circRNA can interact with miRNAs. The circRNAs and mRNAs with common miRNA target sites compete for miRNA binding and form a complex network of interaction and regulation, commonly known as the ceRNA network [14]. Herein, we used bioinformatic tools to predict possible miRNAs that circRNA may bind with. Interestingly, it was noticed that circRNA may interact with miR-34a, which has been demonstrated to be upregulated in cytokines-treated pancreatic *β*-cell and made a great of contribution to cytokines-induced pancreatic *β*-cell destruction [37]. Thus, this finding gave us a significant hint to confirm the functional mechanisms of circRNA in the development of type 1 diabetes. Meanwhile, the two validated downregulated circRNAs were selected to draw a whole picture of ceRNA regulatory networks. This circRNA-miRNA-mRNA network may provide clues to the regulatory pathways that explain the possible functional mechanisms of circRNAs in cytokines-induced pancreatic *β*-cell destruction in type 1 diabetes.

In addition, circRNA-related mRNAs were further analyzed by DAVID Bioinformatics Resources 6.7, so as to predict and analyze the possible biological functions of the two downregulated expressed circRNAs. The annotation results showed that GO analysis enriched terms such as regulation of transcription and regulation of gene expression and KEGG analysis enriched top pathways included TGF-beta and MAPK signaling pathways. These data implied that it will be much worthy to perform further study in exploring the underlying mechanisms of these circRNAs.

Overall, our findings show that proinflammatory cytokines induce extensive changes in the expression of circRNAs. The study of two selected downregulated circRNAs shows that the inhibition of the two noncoding transcripts could suppress insulin biosynthesis and secretion and promote pancreatic *β*-cell apoptosis, suggesting that they may contribute to cytokine-mediated *β*-cell dysfunction occurring during the initial phases of type 1 diabetes. These data provide a perspective for further functional research of circRNAs in cytokines-induced pancreatic *β*-cell destruction and help to clearly clarify the pathology of type 1 diabetes. The two downregulated circRNAs investigated in the study are most probably not the only circRNAs that contribute to pancreatic *β*-cell damage. Thus, further studies of other changed circRNAs induced by cytokine stimulation should be performed in the future. Meanwhile, further studies will not only be needed to be performed in vitro but also be needed to be performed in vivo.

## Figures and Tables

**Table 1:** qRT-PCR primers used.

circRNA or gene	Forward primer	Reverse primer
circRNA 006029	GAGAGTCCGTCGTGTCGTT	AACTTTCACTTGTTTCCCTTCTA
circRNA 013053	GAGCAGGATTCTCTGGTACCT	AGACATCTGTTTCTGTCAAAAGC
circRNA 000286	GAGGCCGGATGATTCTTCTA	TGCAACAGGTGAGTCCTATTG
circRNA 017277	CCTCAGGACTTCCATCGCTTC	CACTCCAGGAAGAGGCGGTC
Proinsulin	TGGCTTCTTCTACACACCCA	TCTAGTTGCAGTAGTTCTCCA
GAPDH	TCTGACGTGCCGCCTGGAGA	CAGCCCCGGCATCGAAGGTG

## Data Availability

The datasets used in the present study are available from the corresponding authors upon reasonable request.

## Abbreviations

circRNA: Circular RNA
ncRNA: Noncoding RNA
miRNA: MicroRNA
NOD: Nonobese diabetic
HCL: Hierarchical clustering
GO: Gene Ontology
KEGG: Kyoto Encyclopedia of Genes and Genomes
ceRNA: Competing endogenous RNA.

## References

[1] Jeck W. R., Sharpless N. E. (2014). Detecting and characterizing circular RNAs. *Nature Biotechnology*.

[2] Memczak S., Jens M., Elefsinioti A. (2013). Circular RNAs are a large class of animal RNAs with regulatory potency. *Nature*.

[3] Meng X., Li X., Zhang P., Wang J., Zhou Y., Chen M. (2017). Circular RNA: an emerging key player in RNA world. *Briefings in Bioinformatics*.

[4] Chen L. L., Yang L. (2015). Regulation of circRNA biogenesis. *RNA Biology*.

[5] Jeck W. R., Sorrentino J. A., Wang K. (2013). Circular RNAs are abundant, conserved, and associated with ALU repeats. *RNA*.

[6] Lu Z., Filonov G. S., Noto J. J. (2015). Metazoan tRNA introns generate stable circular RNAs in vivo. *RNA*.

[7] Nigro J. M., Cho K. R., Fearon E. R. (1991). Scrambled exons. *Cell*.

[8] Zhang Y., Zhang X. O., Chen T. (2013). Circular intronic long noncoding RNAs. *Molecular Cell*.

[9] Salzman J., Chen R. E., Olsen M. N., Wang P. L., Brown P. O. (2013). Cell-type specific features of circular RNA expression. *PLoS Genetics*.

[10] Guo J. U., Agarwal V., Guo H., Bartel D. P. (2014). Expanded identification and characterization of mammalian circular RNAs. *Genome Biology*.

[11] Hansen T. B., Jensen T. I., Clausen B. H. (2013). Natural RNA circles function as efficient microRNA sponges. *Nature*.

[12] Xu H., Guo S., Li W., Yu P. (2015). The circular RNA Cdr1as, via miR-7 and its targets, regulates insulin transcription and secretion in islet cells. *Scientific Reports*.

[13] Ashwal-Fluss R., Meyer M., Pamudurti N. R. (2014). circRNA biogenesis competes with pre-mRNA splicing. *Molecular Cell*.

[14] Ebbesen K. K., Kjems J., Hansen T. B. (2016). Circular RNAs: identification, biogenesis and function. *Biochimica et Biophysica Acta*.

[15] Dube U., del-Aguila J. L., Li Z. (2019). An atlas of cortical circular RNA expression in Alzheimer disease brains demonstrates clinical and pathological associations. *Nature Neuroscience*.

[16] Hansen T. B., Kjems J., Damgaard C. K. (2013). Circular RNA and miR-7 in cancer. *Cancer Research*.

[17] Eizirik D. L., Colli M. L., Ortis F. (2009). The role of inflammation in insulitis and *β*-cell loss in type 1 diabetes. *Nature Reviews. Endocrinology*.

[18] Zhang L., Gianani R., Nakayama M. (2008). Type 1 diabetes: chronic progressive autoimmune disease. *Novartis Foundation Symposium*.

[19] Donath M. Y., Storling J., Berchtold L. A., Billestrup N., Mandrup-Poulsen T. (2008). Cytokines and beta-cell biology: from concept to clinical translation. *Endocrine Reviews*.

[20] McDaniel M. L., Kwon G., Hill J. R., Marshall C. A., Corbett J. A. (1996). Cytokines and nitric oxide in islet inflammation and diabetes. *Proceedings of the Society for Experimental Biology and Medicine*.

[21] Roggli E., Gattesco S., Caille D. (2012). Changes in microRNA expression contribute to pancreatic beta-cell dysfunction in prediabetic NOD mice. *Diabetes*.

[22] Motterle A., Gattesco S., Caille D., Meda P., Regazzi R. (2015). Involvement of long non-coding RNAs in beta cell failure at the onset of type 1 diabetes in NOD mice. *Diabetologia*.

[23] Ruan Q., Wang T., Kameswaran V. (2011). The microRNA-21-PDCD4 axis prevents type 1 diabetes by blocking pancreatic beta cell death. *Proceedings of the National Academy of Sciences of the United States of America*.

[24] Wang S., Wen X., Han X. R. (2018). MicroRNA-30d preserves pancreatic islet *β*-cell function through negative regulation of the JNK signaling pathway via SOCS3 in mice with streptozotocin-induced diabetes mellitus. *Journal of Cellular Physiology*.

[25] Qi H., Yao L., Liu Q. (2019). MicroRNA-96 regulates pancreatic *β* cell function under the pathological condition of diabetes mellitus through targeting Foxo1 and Sox6. *Biochemical and Biophysical Research Communications*.

[26] Li Y., Deng S., Peng J. (2019). MiR-223 deficiency impairs functional *β*-cell mass. *The Journal of Biological Chemistry*.

[27] Zheng Y., Wang Z., Tu Y. (2015). miR-101a and miR-30b contribute to inflammatory cytokine-mediated *β* -cell dysfunction. *Laboratory Investigation*.

[28] Zheng Y., Wang Z., Zhou Z. (2017). miRNAs: novel regulators of autoimmunity-mediated pancreatic *β*-cell destruction in type 1 diabetes. *Cellular & Molecular Immunology*.

[29] Stoll L., Sobel J., Rodriguez-Trejo A. (2018). Circular RNAs as novel regulators of *β*-cell functions in normal and disease conditions. *Molecular Metabolism*.

[30] Cai H., Jiang Z., Yang X., Lin J., Cai Q., Li X. (2020). Circular RNA HIPK3 contributes to hyperglycemia and insulin homeostasis by sponging miR-192-5p and upregulating transcription factor forkhead box O1. *Endocrine Journal*.

[31] Mattick J. S., Makunin I. V. (2006). Non-coding RNA. *Human Molecular Genetics*.

[32] Panni S., Lovering R. C., Porras P., Orchard S. (2020). Non-coding RNA regulatory networks. *Biochimica et Biophysica Acta, Gene Regulatory Mechanisms*.

[33] Esteller M. (2011). Non-coding RNAs in human disease. *Nature Reviews. Genetics*.

[34] Zhang C., Han X., Yang L. (2020). Circular RNA circPPM1F modulates M1 macrophage activation and pancreatic islet inflammation in type 1 diabetes mellitus. *Theranostics*.

[35] Luo S., Deng M., Xie Z., Li X., Huang G., Zhou Z. (2020). Circulating circular RNAs profiles associated with type 1 diabetes. *Diabetes/Metabolism Research and Reviews*.

[36] Haque S., Ames R. M., Moore K., Lee B. P., Jeffery N., Harries L. W. (2020). Islet-expressed circular RNAs are associated with type 2 diabetes status in human primary islets and in peripheral blood. *BMC Medical Genomics*.

[37] Roggli E., Britan A., Gattesco S. (2010). Involvement of microRNAs in the cytotoxic effects exerted by proinflammatory cytokines on pancreatic beta-cells. *Diabetes*.

